# A Comparison of Patients with Hip Fracture, Ten Years Apart: Morbidity, Malnutrition and Sarcopenia

**DOI:** 10.1007/s12603-020-1408-2

**Published:** 2020-06-11

**Authors:** Noelle Probert, A. Lööw, G. Akner, P. Wretenberg, Å.G. Andersson

**Affiliations:** 1 Faculty of Medicine and Health, Örebro University, Örebro, Sweden; 2 Centre of Clinical Research, Region Värmland, Sweden; 3 School of Medical Sciences, Faculty of Medicine and Health, Örebro University, Örebro, Sweden; 4 Department of Neurobiology, Care Sciences and Society, Karolinska Institutet, Stockholm, Sweden; 5 Department of Orthopaedics, Örebro University Hospital, Örebro, Sweden; 6 Department of Geriatrics, Örebro University Hospital, Örebro, Sweden

**Keywords:** Hip fracture, comorbidity, malnutrition, sarcopenia, mortality

## Abstract

**Objectives:**

To investigate possible differences in morbidity, malnutrition, sarcopenia and specific drug use in patients with hip fracture, ten years apart. To analyse 1-year mortality and possible associations with variables.

**Design:**

A prospective, observational study.

**Setting:**

Örebro University Hospital, Sweden.

**Participants:**

Two cohorts of patients with hip fracture, included in 2008 (n=78) and 2018 (n=76).

**Measurements:**

Presence of comorbidity according to the Elixhauser comorbidity measure, multimorbidity defined as ≥3 comorbidities, preoperative American Society of Anaesthesiologists Classification (ASA-class), malnutrition according to the definition by the Global Leadership Initiative on Malnutrition (GLIM), sarcopenia according to the most recently revised definition by the European Working Group on Sarcopenia in Older People (EWGSOP), polypharmacy defined as ≥5 prescribed medications, use of Potentially Inappropriate Medications (PIM) and Fall-Risk-Increasing-Drugs (FRID) and postoperative 1-year mortality.

**Results:**

When comparing the cohorts, significant increases over time was seen for mean comorbidity-count (Difference −1; p=0.002), multimorbidity (Difference −15%; 95%CI −27;−2), ASA-class 3–4 (Difference −25%; 95%CI −39;−9) and polypharmacy (Difference −17%; 95%CI −32;−2). Prevalence of malnutrition and sarcopenia coherently decreased with 22% (95%CI 5;37) and 14% (95%CI 1;29) respectively. One-year mortality remained unchanged and a significant association was found for a higher ASA-class in 2008 (OR 3.5, 95%CI 1.1;11.6) when adjusted for age. Results on PIM exposure suggest a decrease while exposure to FRID remained high.

**Conclusion:**

Our findings support an increasing morbidity within the population over time. However, also presented is a coherent decrease in malnutrition and sarcopenia, suggesting a decrease in frailty as a possible explanation for the observed unaltered mortality, in turn suggesting advances in treatment of comorbidities.

## Introduction

Hip fracture primarily affects older people and low-energy trauma is the most common cause due to osteoporosis and an increased risk of falling. According to Swedish national data the mean age at time of fracture is 82 years and 67% of the patients are of female gender (1). Sweden represents one of the highest incidences worldwide with close to 17.000 cases annually (1, 2). The incidence of hip fracture is estimated to escalate as people live longer (3), a major concern due to the following economic burden, poor outcome and excess mortality (4, 5, 6), 1-year mortality-rate amounting to >25% in Sweden (7).

Patients typically suffer from a high premorbid frailty, multimorbidity and polypharmacy, factors found to increase risk of hip fracture (8, 9, 10). Malnutrition, sarcopenia and comorbidity, overlapping with- and contributing to frailty, being a multifactorial clinical condition (11, 12), are factors associated with an increased mortality post fracture (13, 14, 15). Consensus has recently been reached regarding a definition of malnutrition by the Global Leadership Initiative on Malnutrition (GLIM) (16). Recommendations on defining sarcopenia have also latterly been revised by the European Working Group on Sarcopenia in Older People (EWGSOP) (17). Two major categories of drugs are frequently mentioned in studies. Older people and particularly patients with hip fracture have a vulnerability to Potentially Inappropriate Medications (PIM), associated with increased mortality post-fracture (18). Fall-Risk-Increasing-Drugs (FRID), prevalent within the population, increase hip fracture risk and are also associated with an increased mortality (19, 20). Due to their observed adverse events in older people, several international lists of PIM and FRID have been established in order to increase awareness.

Contradictory to earlier estimates, hip fracture incidence is declining and Swedish data suggests that coherent survival rates have remained unaltered (21), possibly explained by a potential change in morbidity of the population. A few previous studies have examined the development of the population and its morbidity over time and present homogenous results of an increased comorbidity-burden and polypharmacy while mortality has decreased or remained unchanged, possibly portraying advances in treatment of comorbidities, hip fracture and individualized care (22, 23, 24, 25, 26, 27). In light of this there is a value in studying how a possible increase in morbidity may reflect possible changes in malnutrition and sarcopenia as well as specific drug use, to our knowledge not yet studied.

### Aim

The primary aim was to investigate possible differences in morbidity, malnutrition, sarcopenia and specific drug use in patients with hip fracture, ten years apart. Our secondary aim was to analyse 1-year mortality and possible associations with variables.

## Methods

### Study design and population

In this prospective, observational cohort study all patients undergoing surgery at Örebro University Hospital due to hip fracture diagnosed with ICD-10 codes S72.0, S72.1 or S72.2 during 5 months in 2008 and in 2018 respectively, were consecutively invited to participate. No exclusion criteria existed.

### Morbidity and drugs

Data on diseases, ASA-class (28) and medications was obtained from individual medical records. Diseases were verified according to ICD-10, all Elixhauser comorbidities were evaluated (29, 30). Multimorbidity was defined as ≥3 comorbidities. Polypharmacy and excessive polypharmacy was defined as 5–9 and ≥10 prescribed medications respectively.

PIM were identified from indicator 1.1(drugs that should be avoided if explicit reasons for prescription do not apply) of the drug specific indicators compiled by the Swedish National Board of Health and Welfare (SNBHW) (31) and a list (drugs that should be prescribed restrictively) compiled by the Drug and Therapeutics Committee of Örebro County (32). Drugs defined as FRID were identified from indicator 1.8 (drugs and specific symptoms; drugs that increase the risk of falling) by the SNBHW (31) and a list (drugs that can increase the risk of falling) compiled by the Drug and Therapeutics Committee of Örebro County (32). Included drugs can be viewed in Supplementary Dataset S1.

### Malnutrition and sarcopenia

Anthropometric measurements were obtained through clinical bedside examinations.

Malnutrition was diagnosed according to GLIM-criteria (16): At least one phenotypic (listed below) and one etiologic (decreased food intake or inflammatory condition/disease burden) criterion has to be met for diagnosis. Hip fracture was considered an etiologic criterion (16). Phenotypic criteria consist of:

• Low BMI (kg/m2), cut-off < 20 if < 70 years or < 22 if > 70 years (16).
• Reduced muscle mass, measured as calf circumference (CC), cut-off < 31 cm (33).
• Non-volitional weight loss the last three months, measured by the screening-tool Mini Nutritional Assessment (34).

Documentation on weight loss was very poor and therefore excluded from possible phenotypic criteria. Patients were thus considered malnourished if they had low BMI or CC under cutoff in addition to hip fracture as the etiologic criteria.

Sarcopenia was diagnosed according to EWGSOP2-criteria (17), consisting of the following three steps:

• Reduced muscle strength indicating probable sarcopenia. Measured as hand-grip strength using a hand dynamometer, the best attempt of three on the best hand was evaluated, cutoff < 27 kg for men and < 16 kg for women (35).
• Reduced muscle mass confirming diagnosis, measured as CC, cut-off < 31 cm (17).
• Impaired physical performance determining severity; not evaluated in this study.

### Statistics

Differences in mean age, length of stay, comorbidity, BMI, CC and hand grip strength was analysed by independent sample t-test. Differences in gender was analysed by chi-squared test. Level of statistical significance was set at p < 0.05. Differences in proportions for dichotomized variables were calculated with the method described by Newcombe & Altman (36). Differences in proportions are presented as 95% confidence intervals, the interval will be significant if it does not include zero.

Odds ratios adjusted for age were calculated by logistic regression analysis, the 95% confidence interval will be significant if it does not include one.

The t-test, chi-squared test and calculation of odds ratios were performed in SPSS Statistics 25. Differences in proportions were calculated with the software program Confidence Interval Analysis.

## Results

### Participants

In total, 108 patients in 2008 and 97 in 2018 were invited to participate where 30 and 21 patients were unable to, respectively, leaving 78 patients included in 2008 and 76 in 2018. The major reason for non-inclusion was impaired ability to give consent due to cognitive state.

When comparing dropout groups with participants there was no significant difference in gender, in 2008 (p=0.96) or 2018 (p=0.70). In 2008 there was no significant difference in mean age (p=0.26), the drop-out group presenting a mean age of 84 years compared to 81 among participants, whereas in 2018, the dropout group presented a significantly higher mean age of 87 compared to 80 among participants (p=0.007).

### Baseline characteristics

Patients were similar regarding baseline characteristics (table 1), there were no significant differences in mean age or gender distribution. Pre-fractural housing and prevalence of walking aids was similar.

**Table 1:** Baseline characteristics of the two cohorts of patients with hip fracture

	**Cohort 2008 n = 78**	**Cohort 2018 n = 76**	**Difference, [p-value] / (95% CI)**
Age, mean (SD)	81 (11)	80 (12)	−1 year
Min-max	35-98	41-103	[0.55]
Female, n (%)	49 (63)	47 (62)	−1 (−14;16)
Ordinary housing, n (%)	69 of 77 (90)	69 (91)	−1 (−11;9)
Living alone before fracture, n (%)	45 (58)	48 (63)	−5 (−20;10)
Walking aid before fracture, n (%)	33 of 76 (43)	36 (47)	−4 (−19;12)
Length of stay, mean (SD)	10 (5)	9 (4)	1 [0.58]
Coplanar fall-related fracture, n (%)	76 (97)	71 (93)	4 (−3;12)
Fall indoors, n (%)	57 of 71 (80)	53 of 73 (73)	7 (−6;21)
Type of fracture, n (%):			
S 72.0 ^a^	41 (53)	37 (49)	4 (−12;19)
S72.1 ^b^	31 (40)	31 (41)	−1 (−16;14)
S72.2 ^c^	6 (8)	8 (11)	−3(−13;7)


Abbreviations: CI, confidence interval; SD, standard deviation; a. Femoral neck fracture; b. Subtrochanteric femoral fracture; c. Pertrochanteric femoral fracture.

### Morbidity, malnutrition, sarcopenia and drug use

In total, there were 85 comorbidities in 2008 and 133 in 2018. Cohort 2018 presented significantly higher figures of comorbidity, multimorbidity, and ASA-class 3–4. No patients were assessed with an ASA-class higher than 4. Significant differences were seen for the individual comorbidities of uncomplicated hypertension and renal failure, more prevalent in 2018. Pulmonary circulation disorders, complicated hypertension, peptic ulcer disease, AIDS/HIV, blood loss anaemia, fluid and electrolyte disorders, weight loss, obesity, psychoses and drug abuse were not prevalent at all and thus not included in table 2.

**Table 2:** Differences in morbidity, polypharmacy and exposure to PIM and FRID between the two cohorts of patients with hip fracture

	**Cohort 2008, n=78**	**Cohort 2018, n=76**	**Difference, [p- value] / (95% CI)**
Multimorbidity^a^, n (%)	10 (13)	21 (28)	−15 (−27;−2) *
ASA-class 3 and 4, n (%)	27 of 75 (36)	46 (61)	−25 (−39; −9) *
Comorbidity, mean (SD)	1 (1)	2 (1)	−1 [0.002] *
Congestive heart failure	10 (13)	11 (15)	−2 (−13;9)
Cardiac arrythmia	13 (17)	18 (24)	−7 (−20;6)
Valvular disease	3 (4)	5 (7)	−3 (−11;5)
Peripheral vascular disorders	0 (0)	3 (4)	−4 (−11;1)
Hypertension, uncomplicated	20 (26)	42 (55)	−29 (−43;−14) *
Neurological disorder	6 (8)	4 (5)	3(−6;11)
Paralysis	1 (1)	1 (1)	0 (−6;6)
Chronic pulmonary disease	9 (12)	8 (11)	1 (−9;11)
Diabetes, uncomplicated	6 (8)	7 (9)	−1 (−11;8)
Diabetes, complicated	0 (0)	4 (5)	−5 (−13;0)
Hypothyroidism	4 (5)	6 (8)	−3 (−12;6)
Renal failure	1 (1)	7 (9)	−8 (−17;−1) *
Liver disease	0 (0)	3 (4)	−4 (−11;1)
Tumour	6 (8)	6 (8)	−0 (−9;9)
Lymphoma	1 (1)	0 (0)	1 (−4;7)
Metastatic cancer	1 (1)	1 (1)	0 (−6;6)
Rheumatoid arthritis	3 (4)	2 (3)	1 (−6;8)
Coagulopathy	1 (1)	0 (0)	1 (−4;7)
Alcohol abuse	0 (0)	2 (3)	−3 (−9;2)
Depression	0 (0)	2 (3)	−3 (−9;2)
Polypharmacy, ≥5 drugs, n (%)	40 of 77 (52)	52 of 75 (69)	−17 (−32;−2) *
Excessive polypharmacy, ≥10 drugs, n (%)	11 of 77 (14)	16 of 75 (21)	−7 (−19;5)
Number of patients exposed to at least one PIM, n (%)	15 of 77 (20)	11 of 75 (15)	5 (−7;17)
Number of patients exposed to at least one FRID, n (%)	63 of 77 (82)	62 of 75 (83)	−1 (−13;12)


a. Multimorbidity defined as having ≥3 comorbidities of the Elixhauser comorbidity measure; Abbreviations: CI, confidence interval; ASA-class, American Society of Anaesthesiologists Classification; SD, standard deviation; PIM, Potentially Inappropriate Medications; FRID, Fall-Risk-Increasing-Drugs; *, significant.

Polypharmacy was significantly more prevalent in 2018. Results indicate a decrease in PIM-exposure while exposure to FRID remained high. In both cohorts, the most common PIM-categories were hypnotics and sedatives followed by anticholinergics and the most common FRID-categories were cardiovascular FRID followed by psychotropics. Zolpidem was the most frequently prescribed PIM, 10 patients exposed in 2008 and 5 in 2018. In 2008 3 patients were prescribed the PIM Tramadol, not prevalent in 2018. The most common FRID were: Furosemide, Metoprolol, and Zolpidem in 2008 and Furosemide, Metoprolol and Amlodipine in 2018.

**Figure 1A-B:**
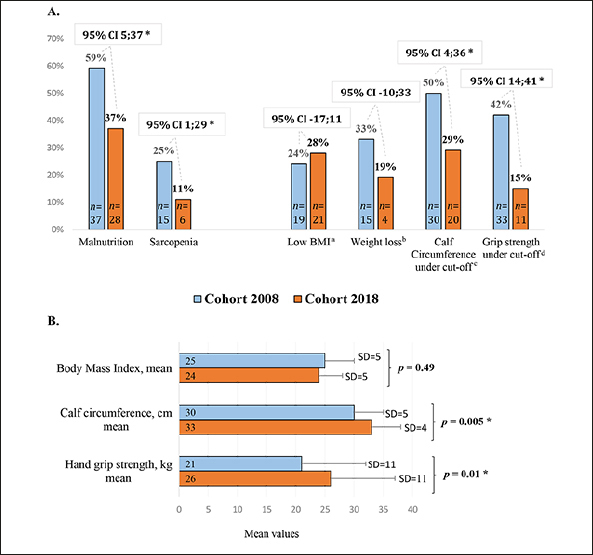
A. Differences in prevalence of malnutrition, sarcopenia, low BMI, weight loss, calf circumference under cut-off and grip strength under cut-off comparing the two cohorts of patients with hip fracture. B. Differences in mean values of body mass index, calf circumference and grip strength comparing the two cohorts of patients with hip fracture Abbreviations: CI, confidence interval; BMI, body mass index; SD, standard deviation; a, kg/m2, <20 if <70 years or <22 if >70 years; b, During the last 3 months; c, <31 cm; d, measured with a hand dynamometer, <27 kg for men and <16 kg for women; *, significant.

Prevalence of malnutrition and sarcopenia was significantly higher in 2008, coinciding with more patients having a grip strength and CC under cut-off (figure 1A). In line with this, cohort 2018 presented significantly higher values of mean CC and grip strength than cohort 2008 (figure 1B). BMI and weight-loss did not differ significantly.

### Mortality

**Figure 2A-B:**
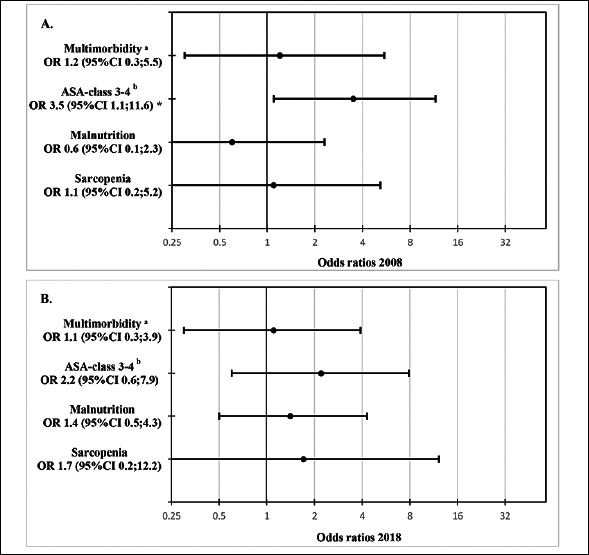
A. Possible associations of variables with 1-year mortality post hip fracture surgery in 2008, presented in a forest plot as odds ratios and 95% confidence intervals adjusted for age. B. Possible associations of variables with 1-year mortality post hip fracture surgery in 2018, presented in a forest plot as odds ratios and 95% confidence intervals adjusted for age Abbreviations: OR, odds ratio; CI, confidence interval; ASA-class, American Society of Anaesthesiologists classification. a, Multimorbidity defined as having ≥3 comorbidities of the Elixhauser comorbidity measure, b, Ranging from 1–6, no patients were assessed with an ASA-class >4; *, significant.

One-year mortality remained unaltered with a rate of 23 % in 2008 and 22% in 2018 (95%CI −13;14). A logistic regression analysis of associations between variables and 1-year mortality was performed (Table 3 and figure 2A-B.), all odds ratios were adjusted for age. Malnutrition and sarcopenia did not present any significant associations with 1-year mortality. For patients with ASA classification 3–4, there was a significant association with 1-year mortality in 2008 (95%CI 1.1;11.6) but not in 2018.

**Table 3:** One-year mortality post-surgery for hip fracture of the two cohorts and possible associations with variables. Odds ratios and 95% confidence intervals adjusted for age

	**Cohort 2008 n=78**					**Cohort 2018 n=76**				
	**Diseased, n =18**	**n**	**Alive, n =60**	**n**	**OR (95% CI)**	**Diseased, n =17**	**n**	**Alive, n =59**	**n**	**OR (95% CI)**
Multimorbidity ^a^	3	18	7	60	1,2 (0.3;5.5)	5	7	6	59	1,1 (0.3;3.9)
ASA-class 3–4 ^b^	10	16	17	59	3,5(1.1;11.6) *	13	17	33	59	2,2 (0.6;7.9)
Malnutrition	6	11	31	52	0,6 (0.1;2.3)	8	17	20	58	1,4 (0.5;4.3)
Sarcopenia	4	11	11	48	1,1 (0.2;5.2)	2	13	4	44	0,9 (0.2;12.2)


Abbreviations: OR, Odds ratio; CI, confidence interval; ASA-class, American Society of Anaesthesiologists Classification; a. Multimorbidity defined as having ≥3 comorbidities of the Elixhauser comorbidity measure; b. Ranging from 1–6, no patients were assessed with an ASA-class >4. *, significant.

## Discussion

### Main findings

When comparing two cohorts of patients with hip fracture from 2008 and 2018, we found a significant increase in morbidity in terms of comorbidity, preoperative ASA-class and polypharmacy. To the contrary, 1-year mortality remained subsequently unaltered and prevalence of malnutrition and sarcopenia significantly decreased.

### Comorbidity and drug use

In consensus with others, patients were of higher age and to a greater extent of female gender. Cohort 2018 presented a significantly higher morbidity in terms of mean comorbiditycount, multimorbidity and preoperative ASA-class. A few previous studies have also compared the population over time and results of a general increase in comorbidity-burden coincide with ours (22, 24, 25, 26, 27). Multimorbidity increased from 13 to 28% (95%CI −27;−2), comparable with the increase of 33.9 to 43.3% found in a large observational study between 2000 and 2016 in USA by Bekeris et al, using the same definition of multi- and comorbidity (25). Significant differences regarding individual comorbidities could only be seen for hypertension and renal failure in this study, suggesting an increase. Although, the potential increase of complicated diabetes mellitus was close to significant (Difference −5%; 95%CI −13;0). These results are supported by Bekeris et al, however, this author found the largest increases in sleep apnea, not reported on in our study as well as in drug abuse, weight loss and obesity, not prevalent in our results (25). This could be attributable to differences in study-sample and design as well as in healthcare and lifestyle between USA and Sweden. Some comorbidities of the Elixhauser comorbidity measure were not prevalent at all in our results, possibly also attributable to small sample-size. Trevisan et al. preformed similarly to us a smaller study comparing a cohort from 2000 with a cohort from 2015 in Italy and also found significant increases in renal disease in addition to alzheimers, COPD, and valvulopathy (26). Significant increases in renal disease, cardiovascular disease and diabetes have also been reported in the larger longitudinal studies by Jantzen et al. from 1999–2012 in Denmark (24), Baker et al. from 2000–2012 in England (27) and Brauer et al from 1986–2005 in USA (22).

Evidently there seems to have been a shift towards increased comorbidity over time. However, there is no way of disregarding possible influences of increased screening, awareness and diagnosis. Also supporting our results though is the coherent increase of ASA-class 3–4 of 25% (95%CI −39; −9), corresponding to preoperative severe systemic disease (28). These findings resemble the increase of 20% found in a population-based study by Turesson et al., observing patients between 1999–2017 in Sweden (37). There are however limitations to ASA-class, the scoring scheme for estimation was revised in 2014, with a re-introduction of case-vignettes (28), possibly affecting assessment. Additionally, the subjective assessment of ASA-class is also a limiting factor, we have tried to diminish this by grouping the ASA-scores of 1–2 and 3–4.

A potential reason for increasing comorbidity could be increasing age of the patients (38). This study presented no significant difference regarding this and results of other studies are inconclusive, Trevisan et al. and Brauer et al. found significant increases in age over 90 and 85 respectively while other studies support our findings (24, 25, 27). Numerous individual diseases have been associated with increased risk of hip fracture (10), therefore a coherent increase in incidence could be expected due to current results on comorbidity. To the contrary, incidence in western countries is constant or declining (39). Causes of this are unclear, the coinciding rise of anti-osteoporotic treatment is a known and debated factor but does not seem to solely explain the situation (40).

Thus, comorbidity seems to be increasing within a decreasing population. A potential explanation could be increasing preventative measures causing the population to exclusively consist of a high-risk profile population, in terms of a higher morbidity. This coincides with the observed increase in polypharmacy (≥5 medications) of 17% (95%CI−32;−2), also being a risk factor (9). Baker et al. found a similar increase of 20%, although defining polypharmacy as ≥4 regular medications. However, studies of the general older population in Sweden have also reported of an increase accordingly and findings in our study could just be reflecting this (41).

Although not significant, results indicate a decrease in PIM-exposure. This resembles decreases seen within the older population in Sweden (42), perhaps bearing witness of increased awareness. Exposure to FRID remained high but psychotropic drugs such as Zolpidem were not as pronounced in 2018 as in 2008, possibly attributable to increased awareness of PIM since these drugs are commonly categorized as both PIM and FRID. Nonetheless, results suggest a lesser awareness regarding FRID, a theory supported by studies reporting of increased prescribing after hip fracture (43).

### Malnutrition and sarcopenia

Since comorbidity has been found a risk factor of frailty and postoperative mortality (11, 13) the main expectation would be that an increase would in turn entail concomitant increases of these conditions/outcomes. Our results however propose the opposite. Frailty, a multidimensional clinical condition increasing with age is predictive of falls, disability, hospitalization and death, thus a major issue concerning the population with hip fracture. Fried et al. came up with a definition of frailty in 2001, since then widely used, including weight loss, exhaustion, weakness, slow walking speed and low physical activity (11). Malnutrition, sarcopenia and weight-loss, also widespread syndromes among older people, interrelate with frailty (12) and can therefore grossly serve as indicators. Interestingly, our results presented significantly higher mean values of hand grip strength and CC in 2018 and patients were less likely to have values under cut-off limits for diagnosis of malnutrition and sarcopenia. BMI did not differ, possibly explained by increased knowledge and treatment regarding nutrition, preserving BMI levels in patients otherwise at risk of malnutrition and weight loss due to disease and concomitant loss of appetite. The documentation of weight loss was very poor, especially for cohort 2018, only reported on in 21 of 76 patients, therefore the diagnosis of malnutrition was only based on CC or BMI under cut-off as phenotypic criteria. Results present a significant decrease in malnutrition of 22% (95%CI 5;37), although possibly underestimated in either cohort since weight loss was not included. Additionally, potential effects of oedema and hereditary traits on CC-value cannot be disregarded, possibly causing overestimation. Due to the GLIM-criteria being new there are to our knowledge no direct comparable studies. The prevalence of malnutrition among patients with hip fracture in other studies varies greatly from <20% to >80%, commonly used criteria are low albumin, vitamin D deficiency, BMI<22kg/m2, weight loss and Mini Nutritional Assessment (44, 45).

Sarcopenia also differed significantly with a prevalence of 25% in cohort 2008 and 11% in 2018 (95%CI 1;29), resembling the prevalence of 17% found in a Spanish study from 2016 using EWGSOP criteria prior to the latest revision (46) and coinciding with the general indication of a decreasing frailty over time. An explanation for a decrease in sarcopenia, malnutrition and possibly frailty could be increased screening, awareness and treatment of the different comorbidities, preventing imminent frailty in older people. Increased awareness as to the importance of physical activity and nutrition is also a possible contributor.

### Mortality

One-year mortality remained unaltered with the rates of 23 % in 2008 compared to 22% in 2018 (95%CI −13;14), in line with results of both Jantzen et al reporting of 9.7% in 1999 compared to 10.3% in 2012 (P=0.9) and Trevisan et al reporting of 25.3% in 2000 compared to 22.2% in 2015 (p=>0.05). In 2008, there was a significant association between ASA-class 3–4 and 1-year mortality (OR 3.5, 95%CI 1.1;11.6) although the association was not significant for Cohort 2018. This could be attributable to increased individualization of healthcare over time, prioritizing those with greatest need. Coinciding with this, Trevisan et al. found significantly worse Charlson comorbidity index scores (a measure of comorbidity) in survived patients 30 days post-surgery in 2015 compared to in 2000. In addition Brauer et al. and a Danish study comparing patients between 1980–2014 (23) found decreases in short-and long-term mortality irrespective of comorbidity-level, suggesting advances in treatment and rehabilitation of hip fracture.

When adjusted for age, we did not find any statistically significant association between 1-year mortality and malnutrition or sarcopenia in this study. Our relatively small cohort size is a limitation, evident when observing results of previous larger studies. For example, a study including 324 patients with hip fracture found that individuals with sarcopenia had a 1.8 times higher 1-year mortality rate than nonsarcopenic (15). Another larger study on 322 patients with hip fracture, showed that malnutrition was an independent predictor of 1-year mortality (OR 2.4) (14). Despite not being able to show it in this study, malnutrition and sarcopenia most likely have a negative impact on survival and the presented decrease in prevalence could be a major factor contributing to the surprisingly unaltered or even decreasing mortality within a population burdened by increasing morbidity.

### Limitations and strengths

A limitation of this study is the small sample-size, decreasing generalizability. Additionally, data regarding comorbidities and drugs were collected from documentation of ICD-10 codes and drugs in medical records that might have caused an over- or underestimation.

The fact that no exclusion criteria existed is also a limitation since pathological and high-energy-trauma caused hip fractures were included as well, however as seen in table 1, 97% versus 93% (95%CI −3;12) had a fracture caused by a coplanar fall.

The dropout group in 2018 had a significantly higher age than the included cohort. Comorbidity is associated with increased age (38), thus results on multimorbidity and medications might have been underestimated in 2018.

The strength of this study is the individually collected data from medical records in combination with individual physical examinations of each patient contributing to a complete evaluation of the population regarding morbidity and frailty in a theoretical as well as a physical sense.

## Conclusions

By comparing two cohorts of patients with hip fracture, a decade apart, our study in line with others suggests an increase in morbidity in terms of increased comorbidity-burden and preoperative ASA-class as well as an increase in polypharmacy. One-year mortality rate remained unaltered and results indicated a subsequent decrease in frailty in terms of malnutrition and sarcopenia.
