# Supplementary material for: A Comparison of Patients with Hip Fracture, Ten Years Apart: Morbidity, Malnutrition and Sarcopenia
Source: J Nutr Health Aging. 2020 Jun 11;24(8):870–7. doi: 10.1007/s12603-020-1408-2 (PMC12876755; doi:10.1007/s12603-020-1408-2)
Supplement: Supplementary file 1 — Supplemental Dataset, S1 [file mmc1.docx]

**Supplemental Dataset, S1**

**Medications included as PIM, presented in ATC-codes**

- Hypnotics and sedatives:
  - Long-acting benzodiazepines: N05BA01, N05CD02 and N05CD03
  - Zolpidem: N05CF02
  - Propiomazine: N05CM06
- Analgesics:
  - Tramadol: N02AX02
  - Codeine: N02AJ06, N02AJ09 and R05DA04
- Anti-inflammatory drugs:
  - Non-Steroidal Anti-inflammatory Drugs: M01A
  - Cox2 inhibitors: M01AH
- Glibenklamid: A10BB01
- Anticholinergic drugs: A03AB, A03BA, A03BB, A04AD, C01BA, G04BD exclusive of G04BD12, M03BC01, M03BC51, N02AG, N04A, N05AA02, N05AB04, N05AF03, N05AH02, N05BB01, N05CF02, N06AA, R06AA02, R06AA04, R06AB, R06AD, R06AE05 and R06AX02

**Medication included as FRID, presented in ATC-codes**

- Psychotropic drugs:
  - Opioids: N02A
  - Antipsychotics: N05A
  - Hypnotics: N05C
  - Sedatives: N05B
  - Anti-depressives: N06A
- Dopaminergic drugs: N04B
- Cardiovascular drugs: C01D, C02-3, C07-9 and G04CA
- Anticholinergic drugs: A03AB, A03BA, A03BB, A04AD, C01BA, G04BD exclusive of G04BD12, M03BC01, M03BC51, N02AG, N04A, N05AA02, N05AB04, N05AF03, N05AH02, N05BB01, N06AA, R06AA02, R06AA04, R06AB, R06AD, R06AE05 and R06AX02

**Abbreviations:** PIM, Potentially Inappropriate Medications; FRID, Fall-Risk-Increasing-Drugs; ATC, Anatomical Therapeutic Chemical Classification System; SNBHW, Swedish National Board of Health and Welfare
